# The neuroexistentialism of social connectedness and loneliness

**DOI:** 10.3389/fnbeh.2025.1544997

**Published:** 2025-07-10

**Authors:** Jamshid Faraji, Gerlinde A. S. Metz

**Affiliations:** ^1^Canadian Centre for Behavioural Neuroscience, Department of Neuroscience, University of Lethbridge, Lethbridge, AB, Canada; ^2^Southern Alberta Genome Sciences Centre, University of Lethbridge, Lethbridge, AB, Canada

**Keywords:** existentialism, social isolation, social enrichment, outreach, stress resilience, MRI, brain plasticity, philosophy of science

## Abstract

Social isolation and loneliness have been subject to extensive investigation and discussion by both modern neuroscience and existentialist philosophy. Neuroexistentialism, though controversial, examines how neuroscientific findings inform human existential concerns. In the present discussion, we argue that (1) in the absence of meaningful attributes, typically provided by relationships with objects and others, social isolation and loneliness lead an individual to a pervasive fear of being or the perception of “*being-in-the-empty-world”* which resembles an existential horror of loneliness; and (2) the pervasiveness of these influences justifies the ubiquity of cerebral responses to both objective and subjective prolonged social disengagement in humans. We also contend that current neuroscientific models of social behaviors, especially within social neuroscience, need to avoid self-affirmative and tautological notions to explain the originality of social connections in human life. By adopting a more integrative and critical approach, these models can better address the complex interplay between social disengagement and their neurological correlates known as the “*social brain*.” This can be accomplished through the establishment of a novel conceptual framework in modern neuroscience to remodel the triad of brain, solitary mind, and society.

## Introduction

Social connectedness represents a critical determinant of brain development and function, profoundly influencing emotional wellbeing, cognitive resilience, and overall health across the lifespan. In ~40,000 adult participants included in the United Kingdom Biobank, loneliness was shown to be associated with a unique neural signature in gray matter volume, intrinsic functional connectivity, and white matter tract integrity, characterizing distinctive structural and functional features of the “lonely brain” (Spreng et al., 2020). These findings imply that the absence of sufficient interpersonal relationships (i.e., social isolation and/or perceived loneliness) detrimentally affects neurobiological integrity. Social isolation is a physical state of being separated from others. Loneliness, however, is an emotional feeling of being disconnected or unfulfilled in social relationships. Both states being alone and feeling alone are pervasive aspects of the human condition, often linked to adverse psychological and physiological outcomes (Cacioppo and Cacioppo, 2018; Faraji and Metz, 2021; Mclennan and Ulijaszek, 2018). Why are human beings so vulnerable to objective or subjective social disconnection? An instantaneous response to the question can be found within the current models and assumptions of the brain-social network domain (Dunbar, 1998; Premack and Woodruff, 2010; Lockwood et al., 2020; Falk and Bassett, 2017; Atzil et al., 2018; Faraji and Metz, 2023; Cacioppo et al., 2015). However, the key answer appears to underline the mind, a hypothetical construct of a functional integration process originating from the brain, in the sense that it provides meaning for identity, along with a theoretical model which emphasizes lived personhood in the leap between brains and social interaction.

Theorizing brain-social dynamics to bridge the gap between brain circuit functions and social behaviors, although revolutionary, seems to ignore how relationship with objects and other people shapes the self and defines identity in a meaningful framework beyond absolute neural function or social connection. The neuroscience of self or identity, therefore, appears to reside within the brain-identity-society ecosystem, where the gap between neural processes and social inputs is filled by the *individual* (i.e., an existential unit; *see below*) and the subjective meaning humans derive from their lived experiences as social animals. Notably, the subjective lived experience of a person accounts for a distinct independent process that forms and delineates identity, based on individual meaning-making within the dialogue between, for example, two persons (brains), as well as the underlying causal neural mechanisms and the broader social dynamics. The notion that humans investigate the meaning of being through relationships with other entities (Abbagnano, 2024), and that the human brain is inherently wired for social connection (Dunbar, 2014), signifies that their capacity for meaning-making is profoundly influenced by their neurobiological architecture, innate tendency toward finding meaning, and social environments. This recognition does not diminish the existential quest for authenticity [Heidegger's German notion of *Eigentlichkeit* (Heidegger, 1927 [1962])] (i.e., acknowledging and embracing individual freedom and transcendence) (Aho, 2023) but rather situates it within a framework that acknowledges the fundamental interdependence of human beings. In the present review, we will discuss the specific domain of existentialism as it pertains to isolation and loneliness, thus exploring their existential reflections through an ontological approach. We will then briefly examine how modern neuroscientific insights inform and transform our comprehension of these profoundly human experiences through an epistemological lens that bridges the objective and subjective realms of isolation and loneliness.

## The solitary existent in search of meaning

A detailed review of existentialism, also known as the philosophy of crisis, is outside the scope of this review. For brevity, it is necessary to omit many arguments irrelevant to the present discussion, even though any condensation and simplification of existential concepts involves certain risks and potential distortion. Existence, as conceptualized by the classic concept of existentialism, is a phenomenon of the inner world of a person which is constituted by the individual's lived experiences and the process of creating meaning and essence through personal choices and actions in relationships with the world (Aho, 2023; Mamedzade et al., 2023). Traditional existentialism emphasizes the intrinsic solitude of human existence and the individual's quest for meaning in a seemingly indifferent universe through a dynamic relationship with objects and others [(Abbagnano, 2024), See also (Kaczanowski, 1962) for further discussion]. Critical situations, from an existentialist perspective, drive a human being to become aware of their existence. This philosophical doctrine underscores the anguish and alienation stemming from the recognition of one's existential isolation.

**Figure 1:**
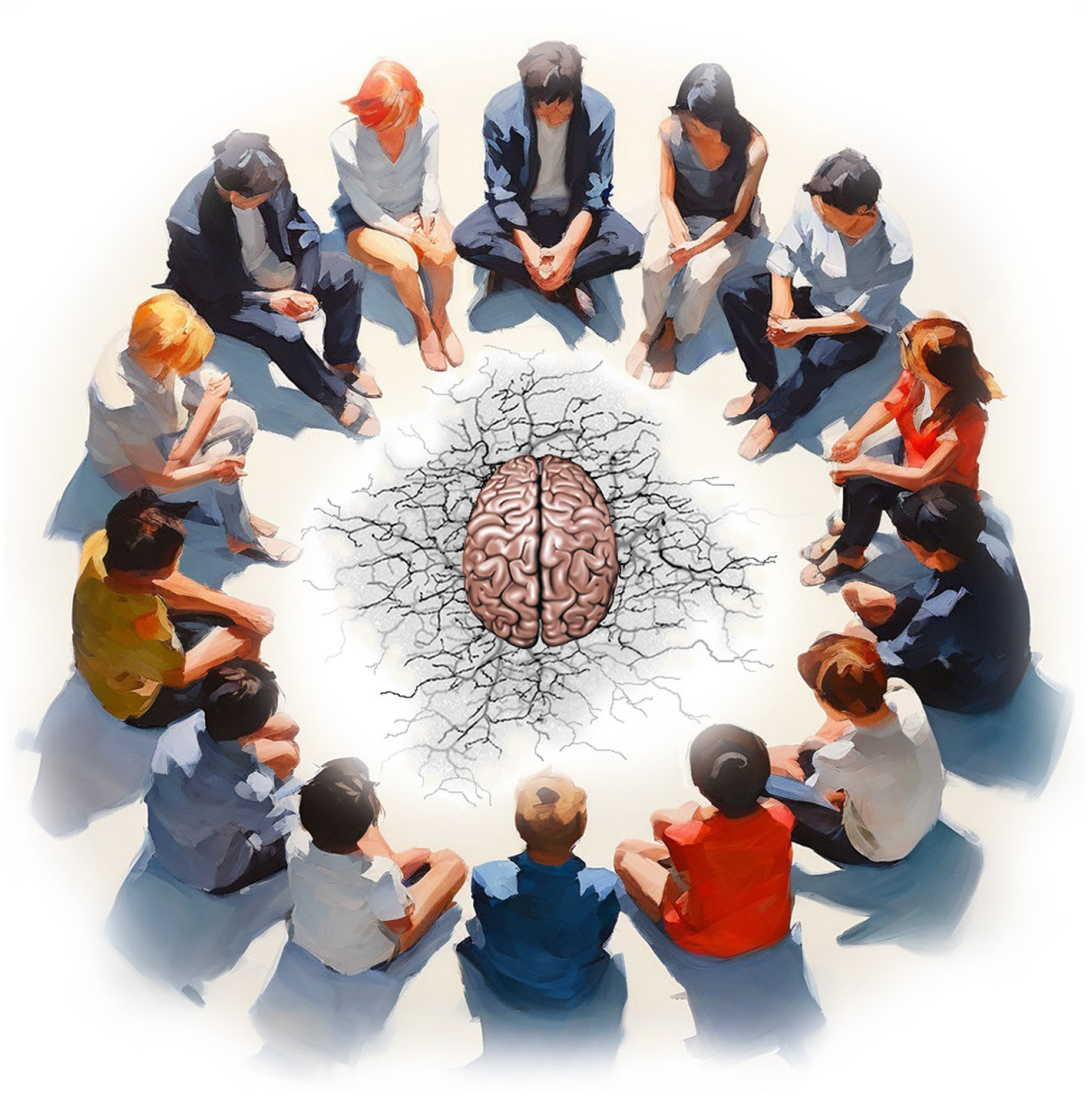
The solitary existent in search of meaning through social connections.

In a very existential interpretation, (i) existence is particular and individual; and (ii) the existent (or individual) encounters the problem of existence, which is primarily the meaning or the mode of being. (iii) In the search for the meaning of being, the existent, however, faces two possibilities: selection (of values) and commitment (to decisions and actions). (iv) Both possibilities involve the existent's relationships with other beings (things and humans), a dynamic that drives the existent to become a “*being-in-the-world*” [*in-der-Welt-sein* (Heidegger, 1927 [1962])], see also (Aho, 2023) for further discussion] which is a meaning-giving activity. (v) Because existence precedes essence, that is, the individual's essence is not given in advance, individuals are forced to create their essence through their choices and actions in relationships. Existentialism, from this perspective, challenges the core concept of any solipsistic doctrine (holding that “I alone exist”), because existence, which is fundamentally constituted by the relationship with other people and objects, always extends beyond itself, toward the being of those entities. Hence, individuals are not confined to their immediate experiences and physical existence. They have the ability to go beyond these limits through their consciousness, choices and actions; it is, so to speak, transcendence or the capacity of individuals to move beyond their immediate circumstances, to engage with the broader world, and to continuously create and recreate their essence through free and conscious choices (Figure 1).

## Isolation and loneliness in an existential frame

The contemporary global situation, characterized by digitalization and an escalation of social and political conflicts, seems critical enough to pervasively impact the individual's existence. Contemporary times encourage a search for the essential truths on which to base the business of living in order to reduce existential tensions (Mamedzade et al., 2023). There are many alarming, typically conflicting changes in the relationships between humans, humans and universe, humans and nature, geopolitics as well as wars and diseases (e.g., pandemics and epidemics) that may profoundly change lifestyle, and increase the complexity and crises of human life (Box 1). Accordingly, conflicting dynamics in social reality intensify human existential tension with the scars of uncertainty, fear, isolation, loneliness, angst and anxiety. Isolation, from an existential perspective, reflects the inherent condition of, physically, being an individual, fundamentally separate from others in one's personal experience and consciousness. It consists of three distinct but intertwined forms: interpersonal, intrapersonal and existential isolation (see Yalom, 1980 for more discussion). However, loneliness, regardless of the variations in its expression (Gallagher, 2023; Shamay-Tsoory and Kanterman, 2024), arises from the existential recognition of this separation, coupled with a longing for meaningful connections and understanding that seem unattainable. This mode of loneliness highlights the tension between the desire for authentic relationships with the world and the inevitable solitude of the human condition. Nevertheless, although conceptually distinct from each other, both “being alone” and “feeling alone” go through similar processes to reflect the same existential dynamics (McKenna-Plumley et al., 2023; Faraji and Metz, 2021).

Box 1 **How does the existential concept of “*I-Thou* vs. *I-It*” explain the terror of loneliness?**

Introduced by the Austrian existentialist philosopher, Martin Buber (Buber, 1958), *I-Thou* (*Ich-Du*) and *I-It* (*Ich-Es*) provide a conceptual framework for understanding human relationships and the terror of loneliness through an existential lens. In an *I-Thou* relationship, individuals engage with each other in a profound, authentic, and mutual way. This connection is characterized by genuine presence, empathy, and reciprocity. Accordingly, in the *I-Thou* relationship, the other person is not seen as an object but as a unique, autonomous being with whom one can have a meaningful interaction. Such relationships are essential for existential fulfillment because they affirm one's existence and provide a deep sense of belonging and purpose. The *I-Thou*, therefore, encounter transcends the ordinary and opens a realm of shared being. In contrast, an *I-It* relationship treats the other person as an object or a means to an end. This interaction is utilitarian and instrumental (Aho, 2023), lacking depth and mutual recognition. The other is perceived as an “*it*” rather than a “*thou*.” Engaging predominantly in *I-It* relationships leads to a sense of isolation and alienation. The individual is deprived of genuine connections, reducing interactions to mere transactions and reinforcing feelings of separateness and loneliness. The terror of loneliness arises from the absence or scarcity of *I-Thou* relationships. Without these meaningful connections, individuals feel isolated and disconnected from others. This profound loneliness is not just physical but existential, a deep-seated sense of being alone in the universe. Hence, loneliness in this context is more than just the lack of social interaction; it is an existential condition. It reflects a profound disconnection from the essence of being, where one feels unseen, unheard, and unacknowledged. The terror stems from the realization of one's fundamental aloneness and the seeming impossibility of bridging the gap between oneself and others. With the exposure to the *I-Thou* relationship, however, “the barriers of the individual are breached” and the existence can create an affective union, representing a “bridge from self-being to self-being across the abyss of dread” (Buber, 1965). In sum, the existential terror of loneliness underscores the human yearning and quest for authentic connections. It reflects the fear that one's existence may remain unvalidated and unnoticed, an existential emptiness which leads to a sense of insignificance and existential dread.

Isolation and loneliness are significant triggers for existential tensions (McKenna-Plumley et al., 2023) because they confront individuals with the inherent meaninglessness of life, especially when crises in social reality persistently restrict or diminish social contact and networking. The existence (life), in an existential discourse, has no inherent meaning, and it is up to individuals to create their own purpose (Mamedzade et al., 2023; Aho, 2023; Gallagher, 2023) merely through being-in-the-world. Isolation and loneliness strip away social roles and external distractions, leaving the existent (individual) to face the stark reality of their existence. Hence, during isolation or loneliness, the absence of imposed meaning from societal interactions forces individuals to confront this void directly, often leading to a sense of despair, alienation, and confusion. Importantly, both isolation and loneliness leave the existent with the absence of external validation (Abbagnano, 2024). Human beings often derive a sense of identity and purpose from their relationships and societal roles. Loneliness removes these external sources of validation, leaving individuals to grapple with their self-worth independently. Although reliance on external validation is an inauthentic way of being, the sudden and persistent lack of this validation can lead to an existential turbulence as individuals struggle to understand their value and significance without external affirmation. Also, individuals are free to make their own choices and must bear the responsibility for the consequences (Aho, 2023). When isolated, therefore, individuals can no longer attribute their choices to social influences or norms, making the weight of their freedom and responsibility more pronounced. This can lead to anxiety and existential angst as individuals realize the full extent of their autonomy and the responsibility that accompanies it. Both isolation and loneliness can also act as catalysts for self-discovery and authenticity. Without the influence of others, individuals have the opportunity to explore their true selves and make choices that are genuinely their own. However, this process can be unsettling and lead to an existential turmoil as individuals question their past decisions, beliefs, and the authenticity of their previous existence (Abbagnano, 2024). Further, both isolation and loneliness magnify the sense of absurdity—the conflict between humans' desire to find inherent meaning in life and the indifferent or empty universe that offers none—as individuals are left to ponder the lack of inherent purpose in life without the distractions of daily social interactions. This confrontation can lead to feelings of futility and existential despair (Mamedzade et al., 2023; Aho, 2023; Dura et al., 2022). Accordingly, isolation and loneliness, from an existential viewpoint, remove the external structures and distractions that often shield individuals from confronting the deeper existential questions about meaning, purpose, and self. This confrontation can lead to an existential tension, characterized by feelings of despair, confusion, and a profound sense of being alone in a meaningless universe.

Thus far, we have discussed how disengagement from social ties and participation, along with a prolonged lack of sense of belonging or engagement with others, leads to a painful experience characterized by existential angst. This angst arises from the struggle to find the meaning of being within the “being-in-the-world” dynamics due to the persistent period of isolation and loneliness. This explanation aligns well with an existentialist interpretation, which opens avenues for incorporating the concept of a *solitary mind* into the brain-social network. As neuroscientists, it is imperative that we develop a more comprehensive understanding of brain function and mental processes by integrating these existential concerns into our field (Box 2). Though controversial (Gabriel, 2018), the dialogue between neuroscience and existential philosophy has recently given rise to the concept of neuroexistentialism (Caruso and Flanagan, 2018), which explores the implications of neuroscientific findings for human self-understanding, freedom, and meaning. By doing so, we can better address the complexities of human experience and improve our approaches to mental health and social wellbeing. More importantly, in an increasingly fragmented and individualistic society, the epoch of digitalization of social relationships and profound alterations in social reality, existential concepts and models are becoming more prominent. Insights into the impact of these existential concerns on brain function and social behavior (Quirin et al., 2012; Wilson et al., 2014; McKenna-Plumley et al., 2023) can inform interventions and policies which are aimed at improving societal mental health.

Box 2 **An epistemological perspective: *The Humanization of Neuroscience*.**

**Figure 2:**
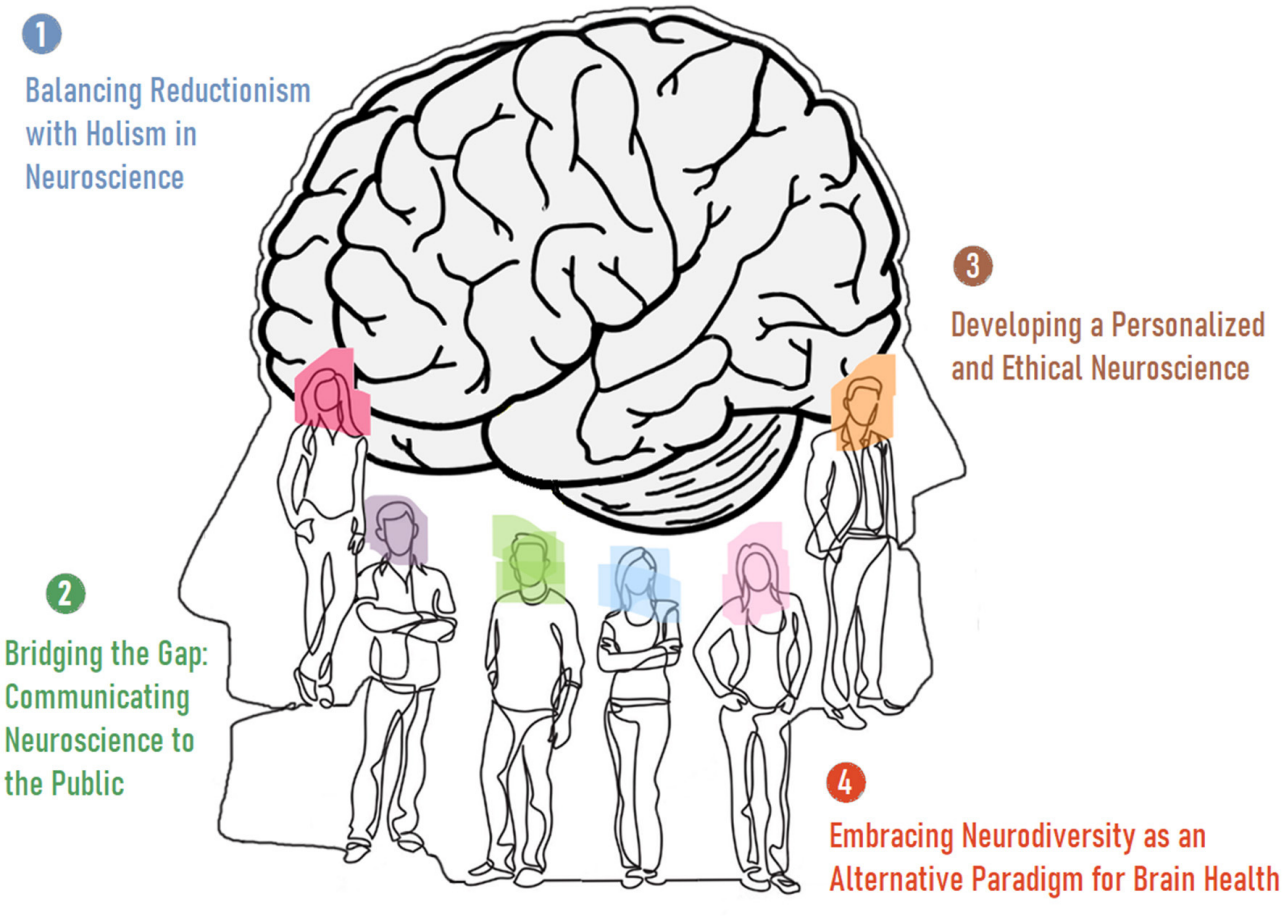
The four fundamental requirements of the humanization of neuroscience.

Neuroscience traditionally focuses on examining the biological mechanisms underlying brain function. The primary aim of these explorations is to provide the best causal explanations of neurodynamics. This mechanistic approach, however, may underestimate the rich subjective experiences that define human life and/or are integral—perhaps even defining—parts of what makes us human. Beyond ethical and social considerations, the necessity of incorporating a multidisciplinary perspective and integrating subjective experiences with objective data is critical for humanizing modern neuroscience. Additionally, neuroscientists must address at least four fundamental concerns (Figure 2) in the realm of empirical/causal insights on brain function: *Limitations of Reductionism*. Reductionist approaches have been highly successful in neuroscience. However, humanizing the field involves acknowledging their limitations, also. Neuroscience should adjust current findings to the premise that complex human behaviors and experiences cannot always be fully explained by dissecting neural components. It also needs holistic and systems-level models that consider the brain's interactions with the body systems and the environment. *Neurodiversity and Neuroinclusivity*. Neuroscience must move away from a one-size-fits-all model of brain function and health by valuing different ways of thinking and being. This approach also includes considering how societal structures can be adapted to better support diverse neurological experiences, whether they are considered normal or abnormal. *Communicating Neuroscience to the Public*. Outreach and effective communication of neuroscience research to the public is crucial for its humanization. Neuroscience should make complex scientific findings accessible and relevant to everyday life. This objective can only be achieved through engaging with diverse communities to ensure that research agendas reflect a wide range of human experiences and concerns. *Personalized Medicine and Research*. Humanizing neuroscience involves tailoring research and medical approaches to individual differences. We need to recognize the variability in brain structure and function across different populations and individuals by developing personalized treatment plans for neurological and psychiatric conditions based on a person's unique neural and genetic profile. Taken together, the humanization of neuroscience is a theoretical challenge that calls for a more integrative, ethical, and person-centered approach to the study of the brain. It requires neuroscientists to look beyond the laboratory and consider the broader implications of their work, bridging the gap between biological mechanisms and the richness of human experience. This challenge is not only about advancing scientific knowledge but also about ensuring that this knowledge is applied in ways that enhance human wellbeing and respect human dignity (Stenning and Bertilsdotter-Rosqvist, 2021; Chellappa, 2023; Krakauer et al., 2017; Subbiah, 2023; Jacobs, 2023).

## Loneliness-induced cerebral dynamics: where the solitary mind meets the brain?

Indeed, there is not only a risk of oversimplifying complex existential concepts when discussing existentialism briefly, but also an unwarranted reductionist dismissal of these existential complexities if one attempts to explain all existential turbulence (such as loneliness) solely through elementary neural processes. We are aware that neuroscientists do not intentionally seek to overlook the importance of such inexcusable flaw. The mind is not the brain, even though mindedness cannot exist without brain circuitry. While it is causally dependent on neural mechanisms, person-specific meaning as a function of solitary mind (as reflected by existentialism) does not appear to be sufficiently reducible to mere neural processes, nor explainable only within a purely social context (Brendel, 2000; Krakauer et al., 2017; Faraji and Metz, 2023). Despite the remarkable progress observed in the study and discourse of most realms of modern neuroscience, including molecular, behavioral, and systems neuroscience, the fundamental neural correlates of existential experiences and thoughts remain incompletely understood. Nevertheless, there is still significant potential to incorporate neuroscientific approaches into existentialist readings of human existential tensions. Firstly, neuroscience can, to some extent, illuminate the biological underpinnings of existential experiences and provide a more comprehensive understanding of phenomena such as meaning-making, anxiety, despair, and loneliness (Mwilambwe-Tshilobo et al., 2019). Secondly, by integrating neuroscientific findings, existentialist frameworks can develop more nuanced therapeutic strategies that address both the physiological and psychological dimensions of existential distress. For instance, functional magnetic resonance imaging (fMRI) has revealed (Quirin et al., 2012) that patterns of neural activation elicited by the threat of death or ultimate limitation, one of the fundamental human existential concerns (Yalom, 1980), are more pronounced than those elicited by the threat of physical (dental) pain. Specifically, these heightened neural responses to the threat of death are observed in the right amygdala, left rostral anterior cingulate cortex (ACC), and right caudate nucleus. Because these limbic subsystems are shown to be linked to implicit or unconscious feelings rather than to conscious emotion (Greenberg et al., 2003; Lane, 2008), the finding may lead to a neuroscientific insight into an existential concern where conscious and unconscious emotional neural processes interact and potentially influence one another in shaping human experience and behavior. More importantly, these conclusions may pave the way for an interdisciplinary cooperation and explanatory pluralism (Brendel, 2000), as opposed to materialistic eliminativism, which denies the significance of causally meaningful psychological states in neuroscientific explanations. However, what would the neural representation of social pain (e.g., rejection) be, given its ontological distinction from an existential tension such as loneliness or threat of death? Research using fMRI has shown that social rejection, such as when individuals contemplate recent rejection by their partners, shares a common somatosensory representation with physical pain in the secondary somatosensory cortex and dorsal posterior insula (Kross et al., 2011). Therefore, it appears that neuroscience can decode and delineate all three levels of human experience—existential, psychological, and physical—each necessitating distinct emotional responses to sociophysiological demands.

It is true that existential loneliness (solitary existence), psychological loneliness (prolonged feeling of being alone) and social rejection (transient feeling of being alone) are conceptually different. Despite the conceptual and practical distinctiveness of these experiences, chronicity (frequency) and depth (intensity) of influences of such emotional experiences along with their neurological representations can be overshadowed by the solitary mind and its quest for the meaning of being. The reason is that the human brain cannot endure a purposeless existence, as all its systems are designed to support meaningful thought and action (Klinger, 1998). Even people who experience social isolation or feel socially rejected enter a state of cognitive deconstruction, characterized by a decrease in meaningful thought (Twenge et al., 2003; Stillman et al., 2009). On the other hand, among the four basic human existential concerns—*meaninglessness* (the absence of given meaning), *isolation* (solitary mind), *death* (the ultimate limitation), and *freedom* (the responsibility to shape one's life) (Yalom, 1980)—isolation, or the terror of aloneness, transcends the common interpretation of subjective social disconnection. Nevertheless, social isolation and loneliness as explained in the contemporary neuroscience are the embodiments of existential isolation, if an individual fails to assign a given meaning to the ongoing objective or subjective non-social or social disengagement while being alone (Breitbart, 2017). If sense of belonging enhances meaning and predicts perceived meaningfulness of life (Lambert et al., 2013), then it can be expected that isolated and lonely individuals suffer from meaningless life (Stillman et al., 2009), and psychological loneliness represents the horror of aloneness which in turn reflects a meaningless existence. Noteworthy, an existential interpretation of isolation distinguishes between mere existence and true life, highlighting the concepts of meaningless existence and purposeless life.

Being connected to social networks is profoundly correlated with the discovery and maintenance of life's meaning. As a result, the subjective perception of a meaningful life fosters social engagement and aids in maintaining close social connections (Steptoe and Fancourt, 2019). This also informs that loneliness and life meaning are negatively correlated with one another (Mwilambwe-Tshilobo et al., 2019; Lambert et al., 2013). Social networks, however, encompass various forms of interactions, ranging from full engagement in social communication and reciprocal acceptance to impoverished interaction or even sociotoxicity, typically characterized by rejection, enforced isolation, and loneliness (Faraji and Metz, 2023; Cacioppo and Cacioppo, 2018; Yang et al., 2016). Interestingly, nearly all types of social experiences are associated with extensive structural remodeling in the brain's gray and white matter, cortical folding, dendritic branching, synaptic connectivity, and myelination (Lloyd-Fox et al., 2017; Faraji et al., 2018a; Peters and O'Donnell, 2005). However, the cerebral representations of social disconnection (perceived isolation and loneliness) are prominently cortical, and gray matter volume shows a more consistent correlation with loneliness than other brain networks (Spreng et al., 2020). This reflects the abstract and perceptual nature of the isolation and loneliness experience in humans, which calls for higher-order central cognitive processing (Baum et al., 2020). Nevertheless, the emotional and motivational contents which involve subcortical regions such as the amygdala (Wong et al., 2016; Lam et al., 2021), striatum (Cacioppo and Hawkley, 2009) and the hippocampus (Wong et al., 2019) should not be overlooked. Recently, the interaction between cortical and subcortical regions of the brain during loneliness has been extensively discussed under the theory of loneliness as social misalignment (Shamay-Tsoory and Kanterman, 2024) which offers a non-existential perspective on isolation and loneliness. However, it is noteworthy that motivational aspects of isolation and loneliness can increase social-seeking behaviors to restore social alignment and/or homeostasis, and ameliorate the unpleasant state of being isolated (Vitale and Smith, 2022; Cacioppo et al., 2014).

The neurobiological mechanisms underlying brain dysfunction induced by social isolation remain poorly elucidated, likely due to the intricate nature of objective vs. subjective isolation in humans and the translational challenges associated with findings from animal models (Xiong et al., 2023). Of note, social animals exhibit behaviors that indicate social isolation can be perceived in a way similar to loneliness in humans. It seems that social isolation induces a range of neurobiological and behavioral responses that resemble symptoms of distress, anxiety, and depression seen in humans. Rodents, for example, show increased levels of stress hormones (such as corticosterone), changes in neuroinflammatory markers, and altered behavior in response to social isolation (Ambeskovic et al., 2025; Faraji et al., 2017; Lopes et al., 2022). One additional circumstance that supports existential conclusions from isolation-induced changes in animal studies is that controlled laboratory procedures provide environments where external threats to survival or objective sources of fear are absent. Generally, the presence of social contact in laboratory settings is associated with increased lifespan across a range of social species (Matthews and Tye, 2019; Faraji et al., 2018b). Conversely, social isolation and loneliness in animal models evoke profound distress, reflected in heightened anxiety-like behaviors, increased fear responses, and neurobiological changes that arguably parallel aspects of existential horror in humans. While animals may not experience existential horror in the same way humans might conceptualize it, they clearly respond to isolation as an aversive state which could be loosely likened to a form of existential distress. These states appear to provoke a pervasive perception of isolation, activating brain circuits (e.g., the hypothalamic preoptic nucleus) implicated in emotional regulation, reward processing, and physiological needs (Liu et al., 2025). Interestingly, chronic social isolation promotes neural activity in the peptidergic fan-shaped body columnar neurons in *Drosophila* (Li et al., 2021). It appears that social isolation in animals leads to a significant alteration in the process of social homeostasis (Matthews and Tye, 2019) and heightened vulnerability to environmental threats, which, in turn, may resemble the existential dread often associated with human loneliness. Social disconnection in animals also elicits consistent and measurable cerebral responses, primarily involving regions associated with threat detection and emotional processing. Significant alterations in gene expression within brain regions integral to social decision-making, such as the prefrontal cortex, hypothalamus, hippocampus, and amygdala have been shown in rodents in response to social isolation indicating the brain's adaptive response to social stressors in animals (Li et al., 2016; Lopes et al., 2024; Xiong et al., 2023; Liu et al., 2025).

The neural fingerprints of loneliness can also be observed in the intrinsic network architecture of the human brain, where patterns of functional connectivity are examined. First and foremost, within- and between-network connectivity in the brain, specifically those involved in the prefrontal, limbic, and temporal systems predict loneliness (Feng et al., 2019). These connections are crucial for cognitive control, emotional processing, and social perception. Similar approaches to social disengagement have been tested using conditional Granger causal analysis of resting-state fMRI data for different neural networks, such as the dorsal attentional network (DAN), the ventral attentional network (VAN), the affective network (AfN) and the visual network (VN) (Tian et al., 2017). The findings profiled similar between-networks connections in lonely individuals: higher loneliness was negatively correlated with weaker causal flow from DAN to VAN, and with decreased causal flow from AfN to VN. Hence, neural networks can predict loneliness.

It is outside the scope of the present discussion to provide a detailed account of the neuroendocrine regulation of these neurocircuitry changes. Nevertheless, it is worth noting that, in parallel with large-scale network alterations, subcortical hypothalamic structures, particularly the paraventricular nucleus and supraoptic nucleus (Jurek and Neumann, 2018) have emerged as central nodes in the neuroendocrine regulation of sociality. Both regions are primary sites for the synthesis of oxytocin, a neuropeptide critically involved in affiliative behaviors and social buffering, and their activity is modulated by perceived social context. Region-specific action of oxytocin may regulate gene expression, cytoskeletal dynamics, and neurotrophic factor expression, such as brain-derived neurotrophic factor (BDNF), which mediates adaptive neuroplastic changes to social experiences (Faraji and Metz, 2025). By contrast, dysregulation of oxytocinergic signaling within the paraventricular nucleus and supraoptic nucleus has been shown to contribute to altered stress reactivity and social withdrawal, which may mechanistically underlie the disrupted functional connectivity patterns observed in lonely individuals (Faraji et al., 2018a; Knobloch et al., 2012; Zheng et al., 2022; Faraji and Metz, 2025).

Moreover, loneliness is not only negatively correlated with meaning in life, but also associated with dense and less modular connections between the default-mode, frontoparietal network (FPN), dorsal and ventral attention, and perceptual networks (Mwilambwe-Tshilobo et al., 2019). More importantly, patterns of resting-state functional connectivity that represent loneliness are inversely related to those representing meaning in life; lonelier individuals share similar patterns of brain connectivity as those with a low sense of meaning. In the neuroscientific domain, loneliness and meaning in life, though conceptually distinct, are ontologically interdependent constructs (Lambert et al., 2013).

Meaning-making is intrinsically a cognitive-affective process. Accordingly, loneliness determines affective and executive cognitive functions within the FPN, which is implicated in conscious emotion regulation and reappraisal (Etkin et al., 2015). Given the close correlation between loneliness and depressive states, and its influence on cognitive control, one can expect that lonely individuals display distinctive functions of the brain's cognitive control network (CCN), which is regulated by the FPN and the affect-related or default-mode network (DMN) (Gao et al., 2020). Functional connectivity between the inferior parietal cortex (IPC) and the rostral dorsomedial prefrontal cortex (DMPFC) is positively correlated with loneliness during working-memory task performance. As part of the DMN, the rostral DMPFC seems to be involved in self-referential mental inspection (Andrews-Hanna et al., 2010) and altered social cognition (Gilbert et al., 2010). The more positive functional connectivity between the IPC and the rostral DMPFC shows that lonelier individuals display increased regulation of self-referential processing, arguably due to the greater negative self and social cognitive bias in lonely people (Gao et al., 2020). Interestingly, loneliness has been shown to be negatively correlated with supplementary motor area (SMA)-caudal DMPFC connectivity (Gao et al., 2020). Because the SMA involves action control and inhibition in humans (Haggard, 2008), one can conclude that reduced connectivity strength between SMA and caudal DMPFC in lonely individuals was induced by their impaired top-down control function which makes them less capable for controlling their actions when performing goal-directed tasks (Gao et al., 2020).

Beyond within- and between-network correlations, signatures of isolation and loneliness can also be observed in the brain's regional responses. Lonely individuals display reduced volume in insular and prefrontal cortex (PFC) (Düzel et al., 2019; Nakagawa et al., 2015) which in turn indicates reduced myelination and synaptic connectivity and transmission, along with reduced signal efficacy. Furthermore, the structure of self-other representation in the medial PFC (mPFC) follows an intrinsic social categorization that directly reflects social connection and connectedness (Courtney and Meyer, 2020). It appears that individuals who are less socially connected or feel lonelier show altered self-other mapping in the mPFC. When neural responses to self and others were examined during an fMRI scan, loneliness was associated with reduced representational similarities between the self and others. Hence, chronic social isolation, during which people persistently feel socially disconnected, can be reflected in a lonelier neural self-representation (Courtney and Meyer, 2020).

Using magnetic resonance imaging (MRI) and voxel-based morphometry (VBM) to examine the local neural representation of loneliness, it has been shown that higher loneliness is reflected in lower gray matter volume in specific brain regions such as the ACC and insula, which are functionally crucial for processing, expressing, and regulating emotionally and socially relevant information (Düzel et al., 2019). The VBM analysis also shows that lonely individuals exhibit reduced gray matter in the left posterior superior temporal sulcus (pSTS) (Kanai et al., 2012), a region involved in basic social perception. The decrease in gray matter is linked to difficulties in processing social cues visually, indicating that loneliness influences how the brain interprets socially relevant visual information. Social isolation and loneliness, therefore, seem to be associated with reduced gray matter volume in brain regions critical for processing and interpreting social and emotional information, thus impacting both cognitive and emotional contents of meaning-making dynamics in lonely individuals.

## Concluding remarks

The concept of *being-in-the-world* represents an engaged, meaningful, and authentic existence, whereas *being-in-the-empty-world* denotes a disconnected, purposeless, and inauthentic state of being. From an existential perspective, we argue that social isolation and loneliness strip existence of its meaning, which explains why the persistent experience of social disengagement and loneliness has such a profoundly detrimental impact on brain architecture. Existence is horrible and fraught with a multitude of existential concerns, including the horror of aloneness and meaninglessness. It is surrounded by objects and others, however, that may assign meaning to existence and act as distractors to prevent our minds from facing the horrific reality of existence and experiencing the pain of uncertainty. Such experiences, which may be induced by both non-social and social disengagement, require individuals to be left alone with their ambiguities or sense of meaninglessness. The disengaged mind (cautiously, an equivalent term for the existentially solitary mind), when the brain is involved in default-mode processing (or just thinking), has already been investigated (Wilson et al., 2014). Though in a social psychological framework, the study showed that individuals typically did not enjoy spending ~15 min in a room by themselves with nothing to do but think. They exhibited a greater enjoyment of mundane external activities and, notably, many individuals preferred administering electric shocks to themselves over being left alone with their thoughts, expectations, decisions and fears (Wilson et al., 2014). It appears that the existential terror of aloneness compels the brain to define existence within a meaningful framework through purposeful, close engagements with both social and non-social entities. If the process of establishing and maintaining meaning repeatedly fails, the individual's sense of self and purpose deteriorates, leading to a collapse in brain function and loss of resilience. Conversely, it appears that enhanced positive social relationships can provide existential resilience, buffering against the threatening impacts of social disengagement and meaninglessness. Existential resilience encompasses the ability to adapt and thrive by developing a coherent sense of self and fostering meaningful relationships and constructive engagements with both the animate and inanimate world.

## Data Availability

The original contributions presented in the study are included in the article/supplementary material, further inquiries can be directed to the corresponding authors.
